# Acute retinal necrosis: A mini review

**DOI:** 10.3389/fopht.2022.916113

**Published:** 2022-08-22

**Authors:** Florence Hoogewoud, Daniele C. Rossi, Theodor Stappler, Yan Guex-Crosier

**Affiliations:** Hôpital Ophtalmique Jules-Gonin, FAA, Université de Lausanne, Lausanne, Switzerland

**Keywords:** acute retinal necrosis (ARN), herpes simplex, varicella zoster, acyclovir, retinal detachment

## Abstract

Acute retinal necrosis is a rare but potentially devastating disease. Even in the era of modern medicine, retinal detachment is a frequent complication leading to vison loss, as well as phthisis bulbi. Whereas IV acyclovir still remains the standard of care, high doses of valacyclovir with/without additional intravitreal injections of foscarnet have been used. In an attempt to reduce the retinal detachment rate, prophylactic laser treatment and early vitrectomy have been proposed. In this article, we aim to review current diagnostic and treatment modalities.

## 1 Introduction

Acute retinal necrosis (ARN) is a direct retinal infection caused by a virus from the herpes family, namely varicella zoster virus (VZV), herpes simplex type 1 and 2 (HSV-1, HSV-2) and rarely cytomegalovirus (CMV) or Epstein Bar virus (EBV). Clinically, ARN is characterized by the presence of peripheral white, flat, well-demarked patches of retinal necrosis, the extent of which can be variable, and which tends to progress in a circumferential pattern. Retinal detachment (RD) being the primary complication, it was part of the initial description of the syndrome in 1971 (1). Generally poor outcomes justify the search for adjunctive treatments which aim to reduce risk of RD. These range from non-invasive prophylactic laser treatment aiming to create a barrier around the areas of retinal necrosis to invasive treatments such as intravitreal injection of anti-viral drugs and prophylactic vitrectomy with and without silicone oil.

## 2 Epidemiology

ARN is a rare disease which represents about 1.2% of uveitis in tertiary care centers (2). Based on two UK population‐based studies, the incidence of ARN is 1 case per 1.6-2 million population per year (3, 4). Males and females are equally affected (4). The age at disease onset ranges from 0-94 years old (4). However, patients with HSV-1 and-2-related ARN are on average younger than patients with VZV-related ARN (34 vs 51 years old) (5, 6). Some studies even report that for patients younger than 25 years, HSV-2 is the most frequent pathogen (7, 8).

## 3 Pathophysiology

VZV is the most frequent causative agent followed by HSV-1 and HSV-2. CMV and EBV infections are rare (9). Necrotizing herpetic retinopathies are characterized by a wide disease spectrum ranging from moderate ARN to severe progressive outer retinal necrosis syndrome (PORN), the latter depending on the patients’ immune status. Even though ARN usually develops in apparently immunocompetent patients, a certain degree of immune dysfunction can be demonstrated in up to 16% of patients (10). The disease is mainly caused by a reactivation of a previous herpetic infection, but rarely it may also be observed as a primary infection (11). For more than half of cases, ARN occurs in patients without any prior history of herpetic infection (4). However, a recent history of chicken pox or shingles can be found in 20 and 15% of patients respectively and a history of viral meningitis or encephalitis has been reported in 15-23% of the cases (4, 12). Whereas HSV-1 is more frequently associated with a history of encephalitis, a history of meningitis is more suggestive of HSV-2 (9).

## 4 Diagnosis

Classification criteria by the SUN (Standardization of Uveitis Nomenclature Working Group) include a peripheral retinal necrosis with either a positive PCR test on any ocular fluid or a characteristic clinical appearance as reported by American Uveitis Society in 1994 (1): one or more foci of retinal necrosis with discrete borders located in the peripheral retina, (2) rapid progression in the absence of antiviral therapy, (3) circumferential spread, (4) evidence of occlusive vasculopathy with arterial involvement, and (5) a prominent inflammatory reaction in the vitreous and anterior chamber (13, 14).

### 4.1 Signs and symptoms

Patients’ main complaints are a red, painful eye with blurred vision and floaters.

**Figure 1:**
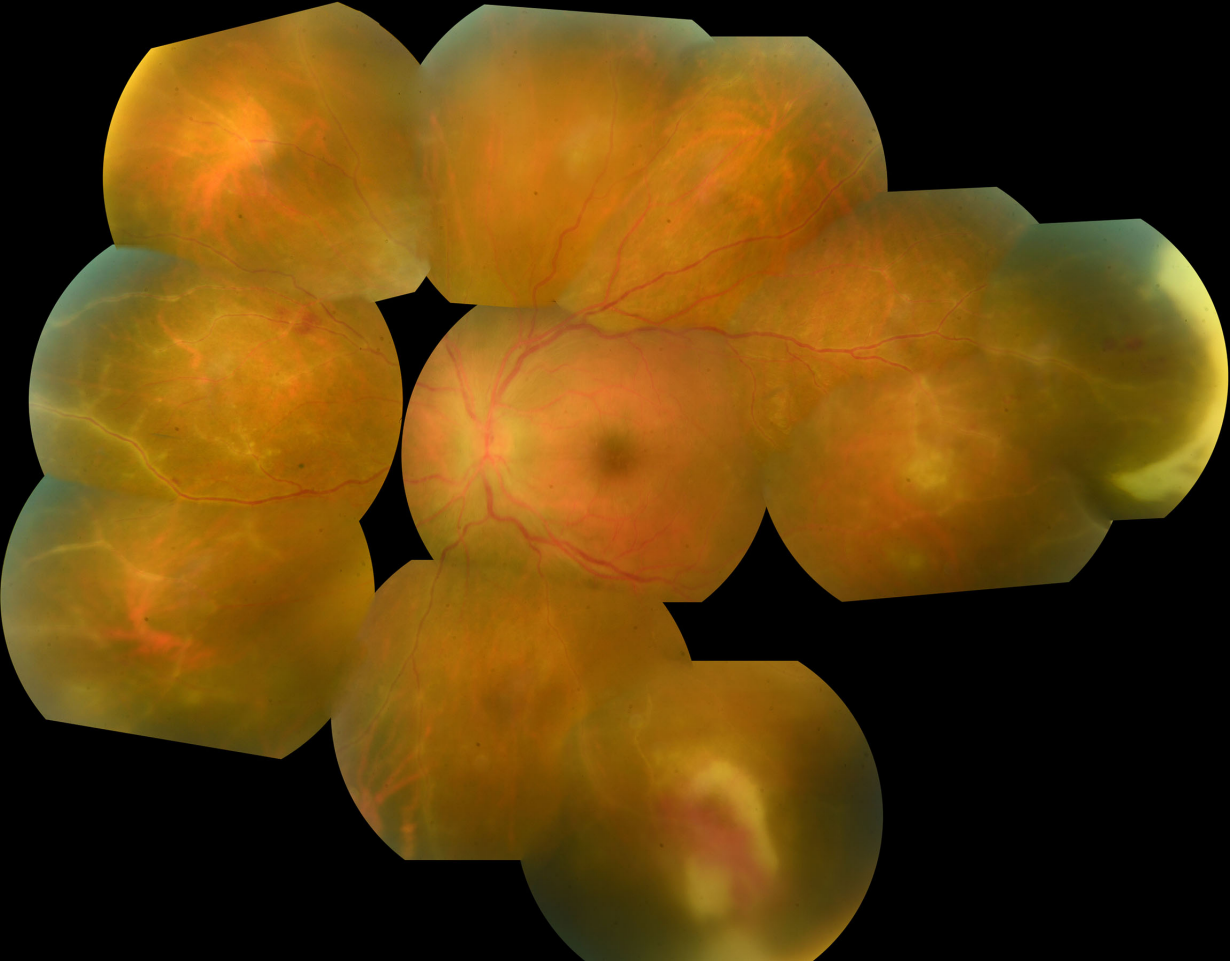
Photo montage of fundus photography of a 25 year old man presenting with large peripheral foci of necrosis associated with severe vasculitis in the presence of VZV infection. Note also the presence of severe vasculitis.

Anterior segment examination typically shows an acute anterior uveitis of variable intensity during early disease stages (15). Concurrent vitreous inflammation is always present in ARN but not in PORN. Typical ARN lesions are located peripherally and appear white-creamy (**Figure 1**). They are well demarcated and tend to progress centripetally towards the center of the eye. The extent of the lesions may range from 2-12 clock hours. It is probably related to the patients’ immune status and the delay before introduction of therapy (15–17). Vasculitis with arteriolar occlusions and hemorrhages might be present (18).

Initial bilateral involvement (BARN; bilateral ARN) is present in 10-15% of cases and may reach 33% during follow-up (3, 4). Contralateral eye involvement usually occurs within 5 months of initial diagnosis but may occur years later (18). The second eye is usually affected less severely and has a lower risk of RD (18).

RD is a common complication of ARN. Wong et al. showed a highly significant correlation between final VA and the presence of RD (p <10^-6^) which makes retinal detachment one of the main limiting factors for a favorable visual outcome (6). Furthermore, this major complication occurs frequently (20% to 85% of treated eyes) thus proving heavy disease burden (19–21). RD occurs usually 3 weeks to 5 months after disease onset (22). Risk factor for the development of RD include low initial VA and greater clock hour extent of the retinal necrosis (23). Also, Wong et al. found a the 2.5-fold greater rate of RD after VZV-ARN (P = 0.013) (6). This data however could not be confirmed in other studies (23).

RD has been identified as a risk factor for subsequent phtisis bulbi which is reported in up to 12.3% of patients (6).

### 4.2 Imaging modalities

#### 4.2.1 Optical coherence tomography

Since most ARN lesions are located peripherally, OCT is of limited use. Nevertheless, in cases of macular involvement, SD-OCT typically shows hyperreflectivity of the inner retina and of the inner plexiform layer (IPL) in particular during early disease. Subsequently, it encompasses the whole retinal thickness (24, 25). Pathophysiologically, the observed hyperreflectivity is presumed to be the result of ischemia secondary to an occlusive vasculopathy of retinal arterioles. The characteristic initial IPL changes could be the result of an occlusive vasculopathy at superficial vascular plexus (SVC) level. This differs from PORN in which the outer retinal layers are involved first (26). As the retinitis progresses, SD-OCT shows disorganization of all retinal layers with the appearance of hyporeflective spaces, due to retinal tissue loss and necrosis (27, 28). At later stages (scar), there is a marked retinal thinning due to retinal necrosis and tractional changes with schisis-like separations (29). Macular edema can also be observed independently from the necrotic lesions (30) owing to intraocular inflammation.

#### 4.2.2 Fluorescein angiography

Fluorescein angiography is helpful in revealing retinal ischemia which might be underdiagnosed during clinical examination (31). Late frames on angiography usually reveal diffuse dye leakage from veins, arteries and/or the optic disc (32). Wide field fluorescein angiography is recommended so as to capture the peripheral retinal lesions.

### 4.3 Quantitative real-time polymerase chain reaction in ARN

Among a multitude of techniques which have been used in the past to detect the pathogen (antibodies in serum and ocular fluids, viral culture, retinal biopsy, and immunocytochemistry) quantitative real-time polymerase chain reaction (qPCR) has become the gold standard for the diagnosis of ARN. Its sensitivity for aqueous and vitreous samples is very high (79-100%) and its specificity is approaching 100% (33, 34). The sensitivity of the test seems to be equivalent if performed on aqueous or vitreous humor and the number of copies of viral DNA are correlated. A direct comparison of the viral copy numbers is however not possible (9, 35, 36). A high number of viral copies (≥5.0 × 10^6^/mL) in aqueous is associated with larger retinal lesions and poor prognosis (lower final VA, higher rate of RD) (37). Quantitative PCR is also useful for the monitoring of treatment response. After an initial plateau phase of varying duration, a logarithmic decrease in the number of viral copies is usually observed. This corresponds to a 50% reduction in viral load within 3 days (38–40). It should be noted that the samples remain positive for a mean time of 56 days after diagnosis, even if the ARN is healed (40).

## 5 Treatment

Intravenous acyclovir has been considered the standard treatment for ARN due to HSV or VZV for 40 years. Its virostatic effect on viral DNA synthesis is derived from the competitive viral DNA polymerase inhibition. Its efficacy in ARN was first demonstrated by Blumenkranz et al. who showed a regression of the lesions under treatment as well as by Palay et al. who concluded to a reduction of the rate of bilateral involvement from 75.3% to 35.1% under treatment (21, 41). The reported 50% inhibitory concentration for acyclovir is 0.02-13.5 μg/ml for HSV1; 0.01-9.9 μg/ml for HSV2 and 0.12 – 10.8 μg/ml for VZV (33). In order for efficacious plasma drug levels to be reached, the current recommended dosage is 10mg/kg given three time a day (as for herpetic encephalitis). The time to peak concentration of intravenous acyclovir (T_max_) is relatively short for plasma (1.5-2-5 hours) as well as for cerebrospinal fluid (CSF)(2.3 hours) (42, 43). Such treatment is expected to lead to a stopping of retinal lesion progression within 48 hours (21).

Since bioavailability of oral acyclovir is poor, valacyclovir, a L-valyl esther prodrug of acyclovir has been developed. This prodrug offers a bioavailability of 54 to 60% (3-5-fold higher than oral acyclovir) with a similar safety profile to IV acyclovir (44). Pharmacokinetic studies show that valacyclovir 2 g TID induced serum levels of 8.49 ug/mL, which are comparable to the serum level obtained with intravenous acyclovir 10mg/kg TID (45, 46). Vitreous drug levels of 1.03μg/ml have been found during vitrectomy, the day after receiving 1g of oral valacyclovir TID (47). The same study describes a mean vitreous-to-serum concentration ratio of 0.24. This ratio is expected to be much higher in the context of ARN due to the major blood-aqueous barrier disruption (48).

The off label use of high dose of valacyclovir 2g TID for the therapy of ARN was first proposed by our team in 2006 and has since been followed by others (49–52). Retrospective comparative studies report similar final VA and RD rate in orally versus intravenously treated patient (53). Some studies even report treatments with lower doses of valacyclovir (1g TID) and favorable responses (51, 52).

### 5.1 Drug resistance and alternative treatments

Drug resistance is rare in VZV but is of growing importance in HSV infections. About 1% of HSV present a drug resistant profile (54). This percentage may rise to up to 30% in immunosuppressed patients with long-term exposure to acyclovir (55). The resistance is due to a mutation in one of two viral enzymes involved in the mechanism of action of acyclovir, namely thymidine kinase (TK) and DNA polymerase, the first one representing over 90% of mutations (55). A mutation of the TK induces a concomitant resistance to famciclovir and penciclovir. Alternatives to treating infection with TK mutations are foscarnet or cidofovir but both must be administered intravenously and have a significant toxicity.

Foscarnet is a pyrophosphate analog which acts on the viral DNA polymerase in a TK independent manner. Current dosage in adult patients is 60mg/kg TID. It has been successfully used in HSV-1 and 2-resistant ARN as well as in ARN due to EBV (56, 57). The safety profile however is not favorable as it can induce severe nephrotoxicity due to tubular necrosis (58). The nephrotoxicity can be significantly reduced with concomitant hydration. It is therefore recommended to administer 750-1000 mL of normal saline or 5% dextrose solution prior to Foscarnet infusion. Due to its multiple drug interactions (e.g. acyclovir sodium, ganciclovir, trimethoprim/sulfamethoxazole, diazepam), its use ought to be carefully weighed.

Cidofovir is used in Foscarnet-resistant herpes viruses presenting a mutation of the viral DNA polymerase. Its efficacy has been reported in case reports (59). The most common adverse effects include nephrotoxicity (12%), and neutropenia (15%) (60).

### 5.2 Intravitreal injections

The use of adjuvant intravitreal injections (IVT) of antivirals (Foscarnet injections (2.4mg/0.1ml) or ganciclovir (200-2000μg/0.1ml) have increasingly been used in recent years (61, 62). They allow a rapid delivery of high doses of medication without inducing retinal toxicity and avoiding the systemic side effects (63). They can substitute the systemic treatment in cases with serious contra-indications, but should preferably be given in addition to it as local treatment alone does not prevent the infection of the contralateral eye. They also present benefits in acyclovir-resistant ARN (64). In ARN patients without systemic contra-indications, its use remains controversial. It is noteworthy that a T_max_ of iv acyclovir in the CSF (and by extension in the eye) can be obtained in as little as 2.3 hours proving that an inhibitory concentration can be reached rapidly. On the other hand, if one extrapolates the data of VZV-related encephalitis, higher doses (15mg/kg TID) of medication may be more effective and therefore the additional local IVT treatment might be favorable to VZV-related ARN (65).

Two comparative studies addressed this question: the first one found a RD rate of 53.6% in the systemic treatment+ IVT group compared with 75.0% in the systemic treatment only group for VZV-related ARN, however, this study did not reach statistical significance (p = 0.23). Neither could this same study reach a conclusion about HSV-related ARN due to small sample size. There was no gain in final visual acuity in either of the virus types or treatment modalities (6). The second study also reports a lower rate of RD in the systemic treatment+ IVT group but the results ought to be viewed with caution since the follow-up in this group has been shorter than in the control group (66).

### 5.3 Adjunctive treatments

#### 5.3.1 Corticosteroids

The role of corticosteroids (CS) in the management of ARN-related inflammation is controversial. Even though comparative studies have not been performed, small case series indicate that the use of oral CS neither influenced the final VA nor the RD rate (62). Small series even reported the use of intravitreal triamcinolone (67). One study assessed the RD rate if CS were given before the diagnosis of ARN and it did not show an increased rate (68). Due to small numbers however, these results should be treated with caution.

#### 5.3.2 Systemic anti-platelet agents

Platelet dysfunction has been demonstrated in 7 patients with bilateral ARN (69). The rationale behind the use of anti-platelet agents like aspirin or warfarin is based on the idea of preventing ischemic events in an environment of occlusive vasculitis. Oral aspirin did not influence the final VA nor the RD rate (62). Again, these case series were too small to prove a beneficial effect.

### 5.4 Preventive anti-viral treatment

After healing of the retinal necrosis, prophylactic long-term oral valacyclovir is recommended for 3-6 months to avoid the risk of relapse or onset of BARN. Prolonged oral therapy >14 weeks was associated with a limited risk of contralateral eye involvement and a better visual outcome (70).

### 5.5 Prophylactic Laser

The purpose of this treatment is to surround the necrotic retinal areas hoping to reduce subsequent retinal detachment. Laser alone however cannot prevent the spread of necrosis and it also requires time to take effect, typically 10 to 14 days, time the patient may not have prior to detachment. Applying “hot” laser burns may also cause retinal holes and, most importantly, its application is heavily dependent on the clarity of the optical media thus excluding patients which may have benefitted from it (6).

Some studies seem to show contradictory results. Lau et al. reports an RD incidence of 35% in the lasered group vs. 80% in the non-lasered group whereas Tibbetts et al. showed an RD incidence of 58% in the lasered and 46% in the non-lasered group (22, 62). A selective bias towards only treating eyes with clearer optical media culminated in another study by Sternberg, where 5 out of 6 eyes were deemed ineligible for laser due to media opacities (71). In summary, such an observable selection bias meant that a good proportion of eyes that could not receive laser. The evidence base for prophylactic laser is therefore inconclusive (72).

### 5.6 Early Prophylactic Vitrectomy

Taken at face value, the rationale for such an invasive treatment is the direct removal of vitreous traction from the retina as well as the removal of the vitreous’ inflammatory load in the process. Pathophysiologically however, retinal detachment in ARN does not primarily stem from unrelieved vitreous traction, but from retinal necrosis and such resulting detachments do not follow Lincoff’s rules for rhegmatogenous retinal detachments making them much more unpredictable. Proponents of prophylactic vitrectomy may point out that such intervention may offer the opportunity to fill the eye with silicone oil, thus preventing future retinal detachment. The presence of an endotamponade such as silicone oil does not prevent the occurrence of retinal detachment, it may limit its spread however, protecting the macula form detaching and allowing the retina additional time to heal. Given the general absence of a natural posterior vitreous detachment in such heavily inflamed, “hot eyes” as well as the additional frailty of a necrotizing retina, the intraoperative induction of a posterior vitreous detachment can only multiply the risk of iatrogenic retinal breaks which, in turn would be prime causes for further retinal detachments themselves.

**Figure 2:**
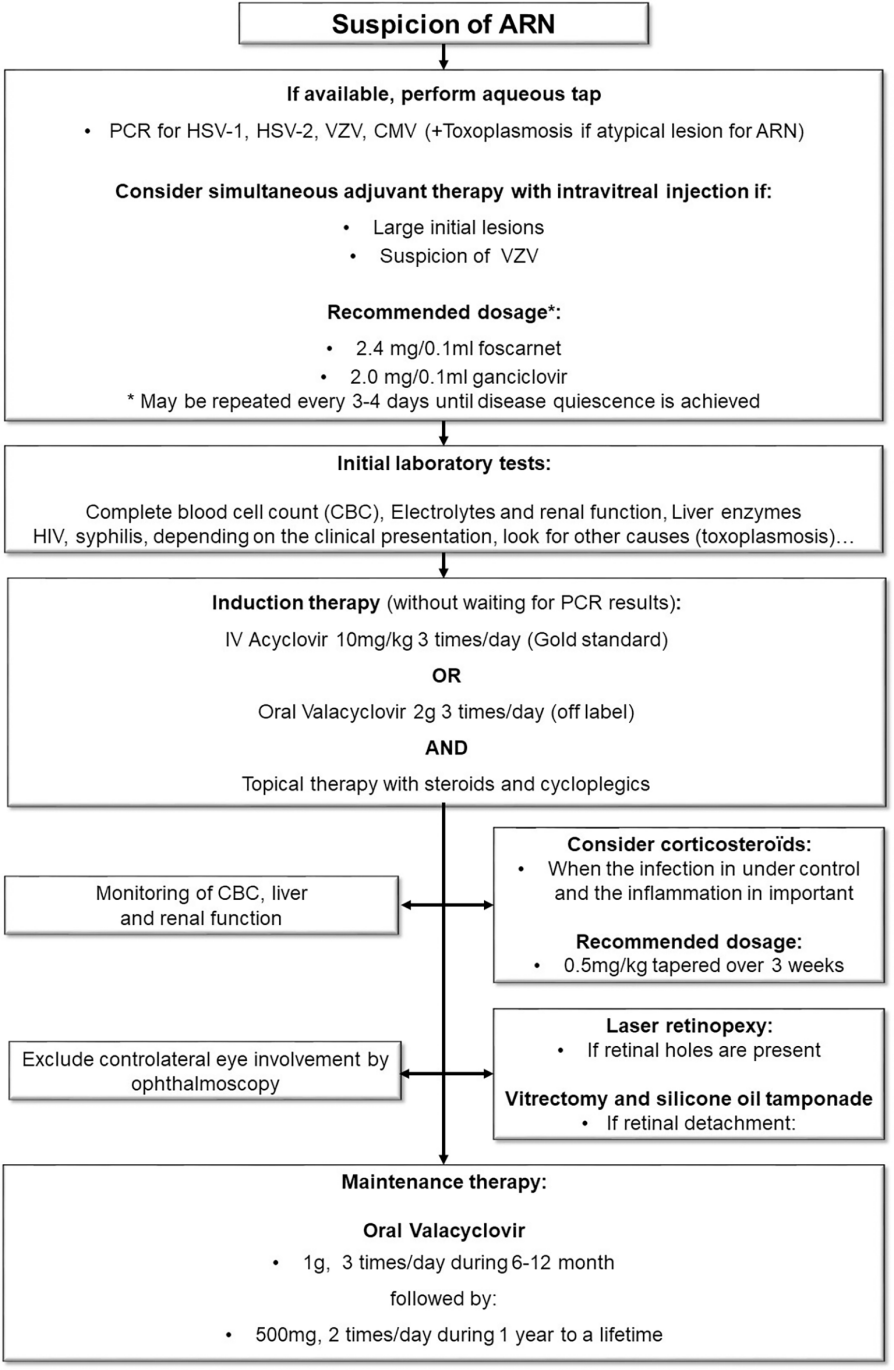
Diagnostic and treatment algorithm.

Hillenkamp et al. compared two treatment regimens before and after 2002 with all patients before that time (n=20) having received only medical treatment and all after that time (n=10) having been treated with early, prophylactic vitrectomy (19). Of the medically treated group 90% went on to develop RD as compared to 40% in the vitrectomy group. This statistically significant difference did not however translate into an improved visual outcome for either group. A larger study by Iwahashi-Shima et al. on 104 eyes with ARN found a final retinal attachment rate of 58% in the early vitrectomy group compared with 75% in the observation group and, again, no anatomic or visual benefit for early PPV could be demonstrated (73). Due to various differences in study design as well as selection bias, neither of the lower-level studies managed to prove a clear benefit of the early vitrectomy approach (74, 75).

### 5.7 Vitrectomy for retinal detachment

Standard procedure for ARN-RD consists of vitrectomy with silicone oil tamponade. The surgical reattachment rate is estimated at 58% which is far below the average retinal reattachment rate for non-ARN detachment. It is also associated with a rate of phthisis bulbi of 12.3% which is far above average for non-ARN pathology (6, 73).

## 6 Conclusion

ARN is a rare but potentially devastating disease. RD is a frequent complication and is associated with a poor outcome. Our current diagnostic and management procedures are depicted in **Figure 2**. Early treatment is one of the main prognostic factors that can be influenced by medical care (17). Whereas qPCR may represent the gold standard for diagnosis confirmation, the clinical suspicion alone should trigger the initiation of treatment without delay. IV Acyclovir remains the standard of care but high doses of oral valacyclovir have equivalent pharmacological properties. The use of Foscarnet IVT is recommended by some authors, but evidence is scarce, especially for HSV-related ARN. After the initial treatment, oral valacyclovir should be used for at least 3-6 month to prevent recurrence.

In summary, this review illustrated the need for further refinement of evaluation criteria and treatment protocols for this rare disease.

## Author contributions

FH, DR, TS, YG-C contributed to the redaction of the review. TS and YG-C have equally contributed as senior author to the supervision of the final manuscript. All authors contributed to the article and approved the submitted version.

## Funding

Open access funding provided by University of Lausanne.

## Conflict of interest

The authors declare that the research was conducted in the absence of any commercial or financial relationships that could be construed as a potential conflict of interest.

## Publisher’s note

All claims expressed in this article are solely those of the authors and do not necessarily represent those of their affiliated organizations, or those of the publisher, the editors and the reviewers. Any product that may be evaluated in this article, or claim that may be made by its manufacturer, is not guaranteed or endorsed by the publisher.
